# Expanded Hemodialysis Therapy Ameliorates Uremia-Induced Systemic Microinflammation and Endothelial Dysfunction by Modulating VEGF, TNF-α and AP-1 Signaling

**DOI:** 10.3389/fimmu.2021.774052

**Published:** 2021-11-11

**Authors:** Rusan Catar, Guido Moll, Julian Kamhieh-Milz, Christian Luecht, Lei Chen, Hongfan Zhao, Lucas Ernst, Kevin Willy, Matthias Girndt, Roman Fiedler, Janusz Witowski, Henning Morawietz, Olle Ringdén, Duska Dragun, Kai-Uwe Eckardt, Ralf Schindler, Daniel Zickler

**Affiliations:** ^1^ Department of Nephrology and Internal Intensive Care Medicine, Charité Universitätsmedizin Berlin, Corporate Member of Freie Universität Berlin, Humboldt-Universität zu Berlin, and Berlin Institute of Health (BIH), Berlin, Germany; ^2^ BIH Center for Regenerative Therapies (BCRT), Charité Universitätsmedizin Berlin, Corporate Member of Freie Universität Berlin, Humboldt-Universität zu Berlin, and Berlin Institute of Health (BIH), Berlin, Germany; ^3^ Berlin-Brandenburg School for Regenerative Therapies (BSRT), Charité Universitätsmedizin Berlin, Corporate Member of Freie Universität Berlin, Humboldt-Universität zu Berlin, and Berlin Institute of Health (BIH), Berlin, Germany; ^4^ Institute of Transfusion Medicine, Charité Universitätsmedizin Berlin, Corporate Member of Freie Universität Berlin, Humboldt-Universität zu Berlin, and Berlin Institute of Health (BIH), Berlin, Germany; ^5^ Department of Cardiology, University Hospital Münster, Münster, Germany; ^6^ Department of Internal Medicine II, Martin-Luther-University Halle, Halle, Germany; ^7^ Department of Pathophysiology, Poznan University of Medical Sciences, Poznan, Poland; ^8^ Division of Vascular Endothelium and Microcirculation, Department of Medicine III, Faculty of Medicine and University Hospital Carl Gustav Carus, Technische Universität Dresden, Dresden, Germany; ^9^ Division of Therapeutic Immunology (TIM), Department of Laboratory Medicine (LABMED), Karolinska Institutet, Stockholm, Sweden

**Keywords:** cardiovascular disease, endothelial cell (dys)function, expanded hemodialysis therapy, chronic kidney disease, end-stage renal disease, uremic toxins / systemic microinflammation, tumor necrosis factor alpha (TNF-alpha), vascular endothelial growth factor (VEGF)

## Abstract

**Abstract:**

Systemic chronic microinflammation and altered cytokine signaling, with adjunct cardiovascular disease (CVD), endothelial maladaptation and dysfunction is common in dialysis patients suffering from end-stage renal disease and associated with increased morbidity and mortality. New hemodialysis filters might offer improvements. We here studied the impact of novel improved molecular cut-off hemodialysis filters on systemic microinflammation, uremia and endothelial dysfunction. Human endothelial cells (ECs) were incubated with uremic serum obtained from patients treated with two different hemodialysis regimens in the Permeability Enhancement to Reduce Chronic Inflammation (PERCI-II) crossover clinical trial, comparing High-Flux (HF) and Medium Cut-Off (MCO) membranes, and then assessed for their vascular endothelial growth factor (VEGF) production and angiogenesis. Compared to HF membranes, dialysis with MCO membranes lead to a reduction in proinflammatory mediators and reduced endothelial VEGF production and angiogenesis. Cytokine multiplex screening identified tumor necrosis factor (TNF) superfamily members as promising targets. The influence of TNF-α and its soluble receptors (sTNF-R1 and sTNF-R2) on endothelial VEGF promoter activation, protein release, and the involved signaling pathways was analyzed, revealing that this detrimental signaling was indeed induced by TNF-α and mediated by AP-1/c-FOS signaling. In conclusion, uremic toxins, in particular TNF-signaling, promote endothelial maladaptation, VEGF expression and aberrant angiogenesis, which can be positively modulated by dialysis with novel MCO membranes.

**Translational Perspective and Graphical Abstract:**

Systemic microinflammation, altered cytokine signaling, cardiovascular disease, and endothelial maladaptation/dysfunction are common clinical complications in dialysis patients suffering from end-stage renal disease. We studied the impact of novel improved medium-cut-off hemodialysis filters on uremia and endothelial dysfunction. We can show that uremic toxins, especially TNF-signaling, promote endothelial maladaptation, VEGF expression and aberrant angiogenesis, which can be positively modulated by dialysis with novel improved medium-cut-off membranes.

**Graphical Abstract:**
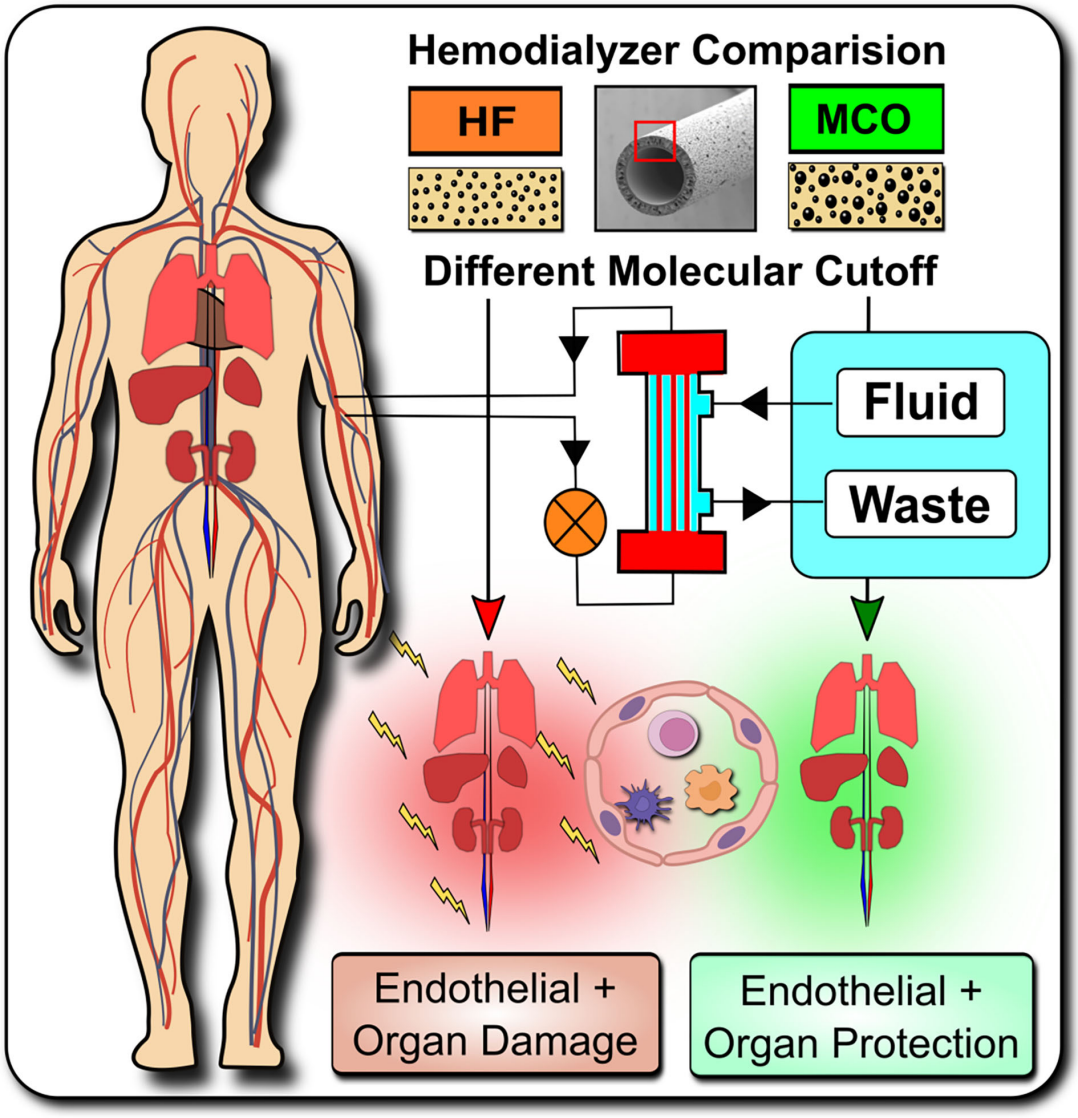
Systemic microinflammation, altered cytokine signaling and cardiovascular diseases are common in hemodialysis patients contributing to the highly increased cardiovascular morbidity and mortality. One of the pathological causes is the endothelial maladaptation and dysfunction associated with uremia and chronic systemic microinflammation. We here elucidate the molecular and biological mechanisms how endothelial maladaptation is induced, and most importantly also how it can be reversed, with in vivo validation in a crossover randomized multi-center clinical study comparing novel improved medium-cut-off (MCO) dialyzers to standard-of-care high-flux (HF) dialyzers.

## Introduction

Kidney disease is a major public health burden (1, 2). The population prevalence of chronic kidney disease (CKD) is ~10% and the portion thereof with end-stage renal disease (ESRD) requiring renal replacement therapy (RRT) is increasing steadily (3). Recently, the growing medical need for RRT has been further aggravated by the Coronavirus 2019 (COVID-19) pandemic (4–7). A popular type of RRT is hemodialysis (HD), an extracorporeal blood cleansing technique that employs dialysis membrane filter-systems to remove toxic metabolic waste products that have accumulated in patients with ESRD (8). Major new research efforts to improve dialysis filters (8, 9), but also to introduce new regenerative approaches (10–12), aim to minimize any undesirable side effects of this important treatment.

Although HD has been effectively employed in the management of ESRD in the past, patients still suffer from considerable side effects, such as greatly enhanced cardiovascular morbidity and mortality (1, 13, 14). In addition to progressive vascular media calcification (15–19), endothelial dysfunction is another key attribute of the cardiovascular disease (CVD) apparent in patients with CKD/ESRD, contributing to the increased morbidity and mortality (20–23). Endothelial dysfunction is thought to result from profound dysregulation of uremic and inflammatory mediators. Due to the complexity of the cellular and molecular crosstalk, the pathomechanisms how these mediators influence the functional outcome remain largely elusive to date and thus need to be explored further in both the chronic and acute setting (24–26).

**Figure 1:**
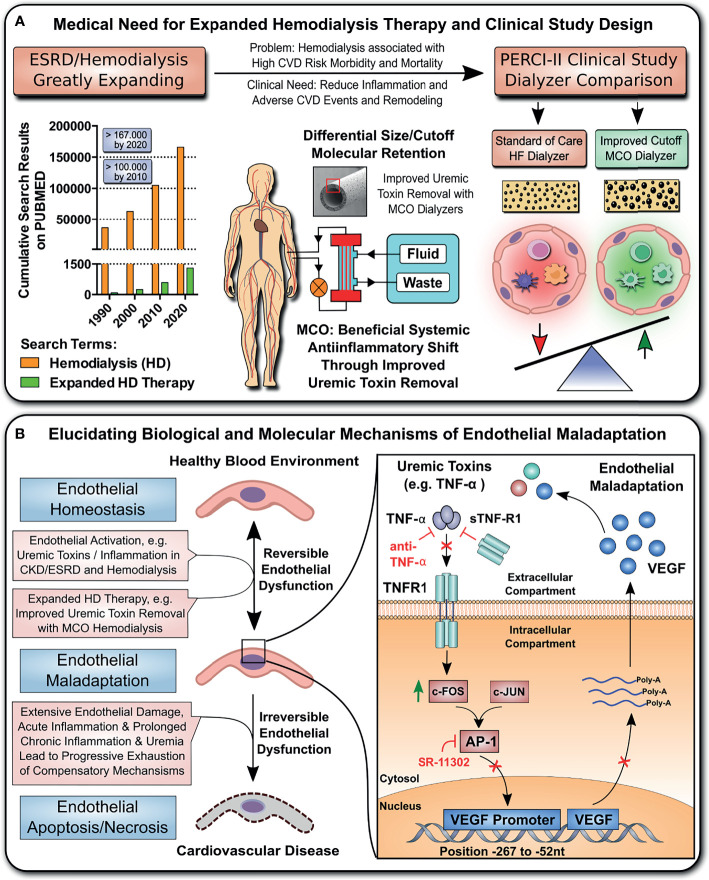
Study design and hypothesis: Expanded Hemodialysis Therapy ameliorates systemic Inflammation and endothelial maladaptation and dysfunction. **(A)** The medical need for Expanded Hemodialysis Therapy and clinical study design: The hemodialysis field has been shown a near exponential growth in the past decades, with >167.000 publications on PUBMED containing the search-term “Hemodialysis” in 2020. Recently, particular attention has been placed into lowering chronic treatment-associated adverse cardiovascular diseases (CVD) and new optimized treatment concepts, such as “Expanded Hemodialysis Therapy” with improved molecular cut-off hemodialyzers (8, 27). Within the PERCI-II study n=48 hemodialysis patients underwent crossover randomized multi-center comparison employing novel medium-cut-off (MCO; MCOI-Ci400, Gambro) dialyzers in comparison to standard of care high-flux (HF) hemodialyzers (PERCI-II-MCO; ClinicalTrials.gov: NCT02084381) (28). These novel MCO dialyzers have an improved molecular size cut-off, which positively modulates systemic microinflammation (28). **(B)** Goal of the follow up study: To Elucidate the Molecular and Biological Mechanisms: In the present study, we explore the molecular signaling mechanisms underlying this positive antiinflammatory shift and evaluate promising leads identified during the first screen in 2017. In particular, we study the modulation of TNF-superfamily members in sera of patients undergoing MCO dialysis and how this impacts on uremia- and TNF-α-induced endothelial maladaptation and dysfunction (left panel) and the molecular mechanisms (right panel), resulting in aberrant VEGF induction and angiogenesis. Our VEGF promoter activation studies and adjunct signaling pathway experiments elucidated that this detrimental uremia- and TNF-α-induced signaling is mediated *via* AP-1/c-FOS signaling and that alterations in the serum ratio between TNF-α and sTNF-R1, but not sTNF-R2, are potential indicators for endothelial maladaptation. These findings provide new avenues for molecular targets and treatment modalities to reduce chronic microinflammation in the context of hemodialysis.

We here elucidate the molecular signaling mechanisms how endothelial maladaptation in response to uremia is induced, and most importantly also how it can be reversed, with *in vivo* validation of these mechanistic findings in a cross-over randomized multi-center study, employing novel medium-cut-off (MCO; MCOI-Ci400; Gambro/Baxter; PERCI-II-MCO study; NCT02084381) dialyzers in comparison to current standard of care high-flux (HF) hemodialyzers (**Figure 1A**). These novel MCO dialyzers have improved molecular cut-off, which positively influences systemic microinflammation (8, 9, 28–30).

Conventional hemodialyzers only eliminate small-sized molecules up to 10–15 kDa, thus leading to a substantial retention and accumulation of numerous middle-sized uremic toxins, such as proinflammatory interleukins and chemokines (9, 28). Hence, long-term HD patients are in a state of chronic systemic microinflammation (21, 24, 30). The new MCO dialyzers with an improved higher molecular cut-off of up to 45 kDa facilitate the effective removal of these middle-sized molecules (8, 9). Furthermore, recent studies have shown a positive impact of “Expanded Hemodialysis Therapy” on systemic microinflammation, but the effect on the vascular endothelium is still mostly unclear to date (9, 28, 31).

The milieu of many chronic inflammatory diseases and in particular HD patients is characterized by disturbed vascular endothelial growth factor (VEGF) production and maladaptive angiogenesis (27, 30, 32–34). Altered VEGF production is associated with an increased morbidity and mortality in HD patients (21–23, 32). VEGF is also a major growth and survival factor for endothelial cells (ECs) and essential for angiogenesis (35–37). Considering proinflammatory inducers/regulators of maladaptive uremic signaling, in particular tumor necrosis factor (TNF) family members, e.g. TNF-alpha (TNF-α) and its soluble receptors (sTNF-R1 and sTNF-R2), have been implied as key agents (20, 24, 27, 30, 38–41). Multiple experts emphasized TNF signaling as central element within the altered cytokine network of uremia-induced EC dysfunction (8, 20, 24, 27, 30). Importantly, soluble TNF receptor levels and their ratios with TNF-α can be interpreted as a marker indicative the severity and harmful effects of TNF-α associated inflammation in different disease states (40, 41).

In this study, we have investigated the effect of HF and novel MCO dialyzer obtained uremic serum on endothelial VEGF production and maladaptive angiogenesis. We identified the role of TNF superfamily members in uremia-induced endothelial activation and adjunct changes in angiogenic homeostasis, with in-depth deciphering of the concomitant signaling pathways. Importantly, the new MCO dialysis membranes with improved porosity were found to positively modulate endothelial maladaptation and dysfunction.

## Methods

### Patient Description, Serum Samples and Multiplex Cytokine Analysis

**Figure 2:**
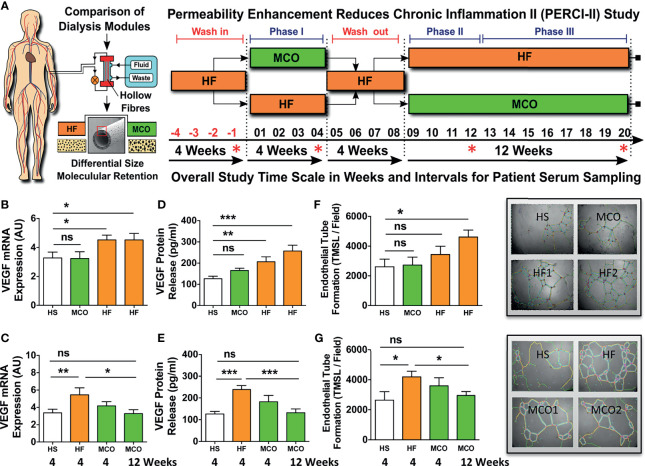
Hemodialysis with improved MCO dialyzers normalizes endothelial VEGF production and maladaptive angiogenesis upon uremic serum exposure *in vitro*. **(A)** Schematics of patient serum collection for analysis of endothelial VEGF expression and angiogenesis/endothelial tube formation after stimulation of ECs with respective sera. Within the PERCI-II study, patients underwent alternating hemodialysis (HD; in 4 weeks intervals) with either high-flux (HF) or medium cut-off (MCO) dialyzers (n=23-25 patients). Upon a 4-weeks wash-in phase on HF dialyzers, patients were allocated for 4 weeks to either HF or MCO dialyzers, followed by a 4-weeks wash-out phase on HF dialyzers, followed by a 12-weeks allocation to HF or MCO dialyzer. The top row shows regimen A (HF, MCO, HF, HF) and the bottom row shows regimen B (HF, HF, MCO, MCO). The sera/time points used for analysis in the second part of the figure are indicated with red stars: end of wash-in, end of phase 1, 2, 3; and **(B, C)** Endothelial VEGF mRNA expression (AU; arbitrary units, 3-hour stimulation; n=23-25), and **(D, E)** VEGF protein release (pg/ml) upon 24-hour stimulation with either 10% HF-HD or 10% MCO-HD serum (n=23-25), and **(F, G)** Endothelial tube formation (TMSL/field; n=23-25) upon stimulated with either 10% HF-HD or 10% MCO-HD serum for 16 hours, as compared to healthy serum (HS) controls. ANOVA, Mean ± SEM, with *P < 0.05, **P < 0.01, and ***P < 0.001. ns, not significant.

**Figure 3:**
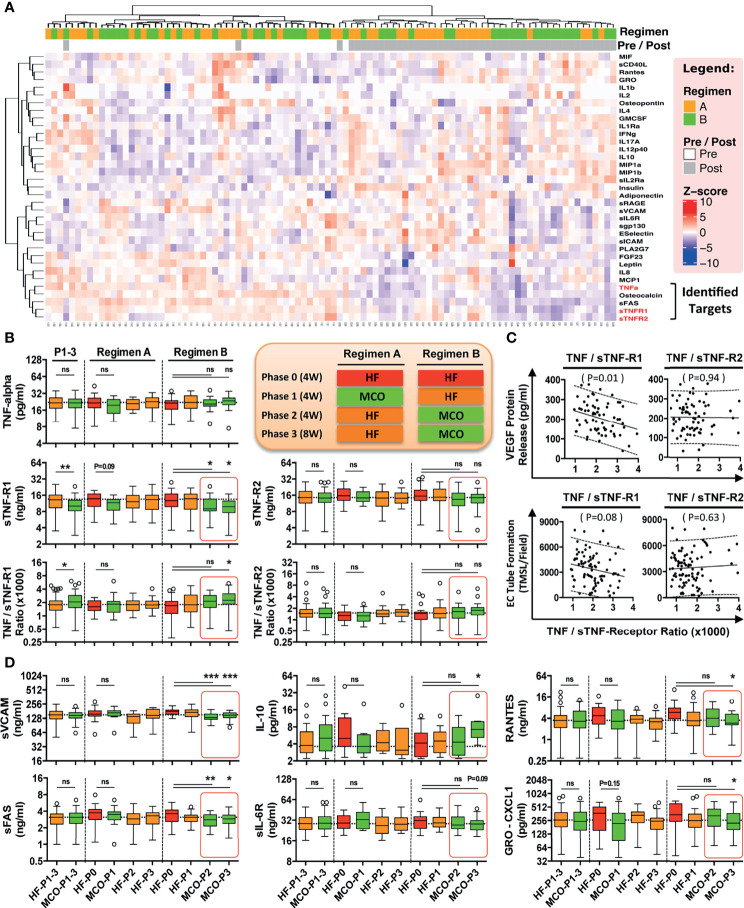
MCO dialysis alters the systemic TNF-α/s-TNF-R1-ratio correlating with endothelial protection *in vitro* and a beneficial shift in serum cytokine levels *in vivo*. The patients underwent the two different hemodialysis (HD) regimes as indicated in (either HF/MCO/MCO or MCO/HF/HF; n=23-25) and the levels of soluble mediators at the different time points were analyzed with multiplex Milliplex and Luminex technology (28). **(A)** Unsupervised clustering heat-map analysis of biomarkers (rows) and patients (columns); **(B)** Quantification of TNF-α, and sTNF-R1 and sTNF-R1-R2 and their corresponding ratios; **(C)** Correlation of the TNF-α/sTNF-R1- and TNF-α/sTNF-R2-ratios with endothelial VEGF release (pg/ml) or endothelial tube formation (TMSL/field) upon stimulation with either 10% HF or 10% MCO patient serum; and **(D)** Patient serum profiling for biomarkers of endothelial activation and systemic inflammation. [Legend to **(B)** and **(D)**] Central legend (simplified depiction corresponding to the clinical trial scheme shown in ) indicates the underlying color code of the samples/time points in the box plots from regimen and the length of their phases (Phase 1 and 2 four weeks and phase 3 eight weeks, abbreviated as P1, P2, and P3 with duration shown in brackets), which show cytokine analysis of serum samples at the end of HF wash-in phase (Phase 0, the standard proinflammatory baseline before start of Regimen A or B which indicated in red), and the end of Phase 1, 2, and 3 (HF shown in orange, and MCO shown in green in legend and corresponding box plots), and the analyzed samples correspond to the end of the phases (Corresponding to the red stars in **Figure 2A**). Each box plot is labeled with the corresponding dialysis filter device (HF or MCO) and the trial stage (P0, P1, P2, and P3), as indicate in the central legend. ANOVA, Box plots Tukey with interquartile range, with *P < 0.05, **P < 0.01, and ***P < 0.001. ns, not significant.

**Figure 4:**
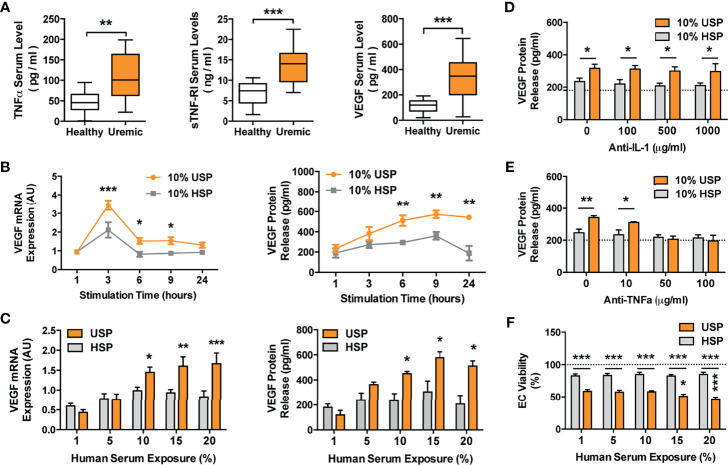
Elevated TNF-α and VEGF levels in uremic serum and VEGF induction in ECs by uremic serum, but omission of VEGF induction by TNF-α blockade. **(A)** Levels of TNF-α (pg/ml), sTNF-R1 (ng/ml) and VEGF (pg/ml), in sera derived from healthy control subjects or uremic hemodialysis patients (n=14), used to generate the healthy and uremic serum pools (HSP and USP, respectively; Mann-Whitney test, Box plots min-max range); **(B, C)** Kinetics and dose-response of endothelial cell (EC) VEGF mRNA (AU; arbitrary units; n=6) and protein production (pg/ml) in response to incubation with HSP or USP (both 2way-ANOVA); **(B)** To assess kinetics of VEGF production, the ECs were incubated for different time points (1-24 hours) with 10% serum with the peak of VEGF mRNA expression detected at 3 hours and maximal protein expression at 6-24 hours; **(C)** To assess the dose-response of VEGF production the ECs were incubated with different concentrations of (1-20% serum) with maximal VEGF mRNA expression and protein secretion being detected in response to 10-20% serum after 3 and 24 hours of incubation respectively; and **(D–F)** The effect of either: **(D)** Anti-IL-1 receptor antagonist Anakinra, or **(E)** Anti-TNF-α blocking antibody Infliximab, on human uremic serum-induced VEGF release in ECs. The cells were pre-treated with or without either Anakinra or Infliximab for 1 hour, followed by stimulation for 24 hours with either 10% USP or 10% HSP (n=7), and ECs were subsequently assessed for VEGF release (both 2way-ANOVA); and **(F)** Dose-dependent effect of 1-20% USP *vs*. 1-20% HSP on EC viability, with assessment of EC viability (% viable cells, n = 6) with the WST-8 cell viability assay after 24-hour stimulation (2way-ANOVA). Box plots min-max range with Mann-Whitney-test, other plots 2way-ANOVA-testing with mean ± SEM, with *P <0.05, **P <0.01, and ***P <0.001.

The uremic serum samples were obtained during the Permeability Enhancement to Reduce Chronic Inflammation-II clinical trial (**PERCI-II-MCO; ClinicalTrials.gov NCT02084381;** https://clinicaltrials.gov/ct2/show/NCT02084381) (28). The study was conducted in accordance with the ethical principles of the Declaration of Helsinki and approved by the Ethics Committees of the Martin-Luther-University Halle-Wittenberg and the Charité Berlin, and written informed consent was given prior to inclusion of subjects into the study. The description of baseline clinical parameters (**Table 1**) for patients starting with either MCO (n=23) or HF (n=25) dialysis shows that both groups had similar age, sex, body-mass-index, dialysis vintage, underlying diseases and lab values (28). The serum samples from 48 study participants were collected at different visits (Visit 1 to 7) after a 4-weeks run-in-phase with HF prior to the first dialysis session. The samples were centrifuged at 2000 g for 15 min, followed by cryostorage at −80°C to be used for the EC incubation assay and multiplex analysis presented in **Figures 2**, **3**. Quantification of soluble markers in patient serum was conducted on citrated plasma using the Milliplex Human Cytokine assay (Millipore) and Luminex-based immunoassays for soluble receptor proteins and specific ELISAs (28, 37). For mechanistic experiments (**Figures 4** **–** **6**), samples of 20 patients were pooled at equal volumes to obtain a uremic serum pool (USP) and non-uremic serum from 14 healthy donors (Age 36 ± 9.2 years; nine males, five females) was collected at our department, to generate a healthy serum pool (HSP) (**Table S1**) (17). All chemicals were purchased from Sigma (St Louis, US) and culture plastics from Becton Dickinson (Falcon; Franklin Lakes, US). Cell culture media and buffers were purchased from Biochrom-AG (Berlin, Germany) and fetal calf serum (FCS) from Invitrogen (Darmstadt, Germany). Recombinant TNF-α and SR-11302 were obtained from Tocris Bioscience (Wiesbaden, Germany), Infliximab from Hospira Inc. (Lake Forest, US), and sTNF-R1 from R&D Systems Inc. (Minneapolis, US).

**Table 1:** Description of patient population at baseline.

	MCO first (n=23)	HF first (n=25)	P-value
**Demography**			
Patients Age (years)	58.1 ± 16.6	59.8 ± 16.5	0.72
Male Patients	n=19 (83%)	n=16 (64%)	0.2
Female Patients	n=4 (17%)	n=9 (36%)	
BMI (kg/m^2^)	27.6 (± 4.1)	27.1 (± 5.4)	0.66
Dialysis vintage (months)	77.3 (± 78)	54.0 (± 50)	0.35 (Mann Whitney)
**Underlying Renal Disease**			
Diabetic nephropathy	3 (13%)	5 (20%)	0.093 (Monte Carlo simulation used because of small cell frequencies)
Glomerulonephritis	4 (17%)	5 (20%)	
Interstitial, Analgetics, Reflux	3 (13%)	2 (8%)	
Cysts	1 (4%)	5 (20%)	
Hypertension, Glomerulosclerosis	6 (26%)	0 (0%)	
Others	6 (26%)	7 (28%)	
**Lab values**			
CRP (mg/l)	13.5 (± 25.1)	9.9 (± 10.4)	ns
Albumin (mg/l)	36.8 (± 3.2)	37.2 (± 3.3)	ns

MCO, medium cut-off; HF, high flux, CRP, C-reactive protein; Entries are absolute and percentage frequencies and means ± Standard Deviations; and P-values refer to two-sided t-tests and chi-square tests if not stated explicitly. ns, not significant.

**Figure 5:**
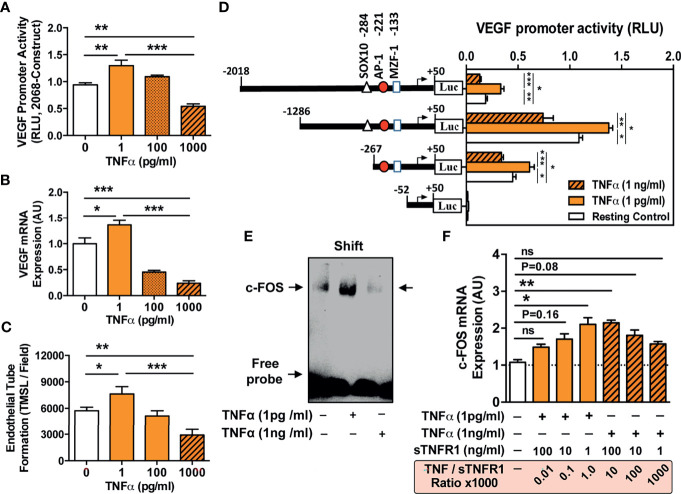
TNF-α concentration-dependent VEGF promoter activation and angiogenesis and VEGF promoter sequences responsive to TNF-α stimulation. **(A–C)** ECs were stimulated with different concentrations of TNF-α (1 to 1000 pg/ml) to assess: **(A)** VEGF promoter activation (RLU; relative luciferase activity, 3-hour stimulation with TNF-α, n=4) in cells transfected with the pLuc 2068 full-length luciferase-reporter construct; and **(B)** VEGF mRNA expression (AU; arbitrary units, 3-hour stimulation with TNF-α, n=4); and **(C)** Endothelial tube formation (TMSL/field; total master segment length per field averaged of 5 assessed fields per condition, 16-hour stimulation with TNF-α, n=12). **(D)** To identify VEGF promoter sequences responsive to TNF-α stimulation ECs were transfected with different VEGF promoter constructs subjected to progressive 5’-deletions (full-length -2018 construct and -1286, -267, and -52 deletion) and the relative luciferase activity (RLU, n=4) determined in cells stimulated for 6 hours with TNF-α (1 pg/mL or 1000 pg/ml) compared to unstimulated resting control cells, demonstrating a loss in VEGF promoter activity upon truncation of the promoter region spanning the positions -267 to -52, and simultaneous identification of a potential high affinity AP-1/c-FOS binding site at position -102 with computation analysis. **(E)** Validation of the AP-1/c-FOS transcription factor-binding site with EMSA (one representative experiment shown) using biotin-labeled double-stranded oligonucleotides targeting to the calculated AP-1/c-FOS positions at -95 to -119 of the corresponding VEGF promoter region. ECs were stimulated for 6 h with TNF-a (1 pg/mL or 1000 pg/ml) and nuclear fractions analyzed for formation of nuclear complexes with the c-FOS probe (labeled as a shift with arrow); and **(F)** Effect of sTNF-R1 (1, 10, and 100 ng/ml) on the modulation of TNF-α induced c-FOS mRNA-expression (AU; 3-hour stimulation with 1 or 1000 pg/ml TNF-α, n=4) with corresponding TNF-α/sTNF-R1 ratios (0.01, 0.1, 1.0, 10, 100, 1000). 1way-ANOVA with Mean ± SEM, with *P < 0.05, **P < 0.01, and ***P < 0.001. ns, not significant.

**Figure 6:**
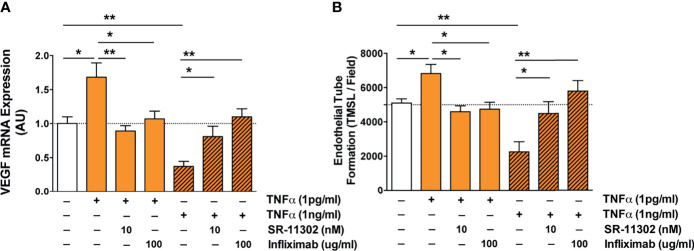
Analysis of the molecular signaling pathways underling TNF-α induced endothelial VEGF induction and angiogenesis identifies AP-1/cFOS-signaling. **(A, B)** Role of the AP-1/c-FOS signaling pathway in low and high TNF-α level-induced endothelial VEGF induction (AU; n=5) shown in **(A)** and *in vitro* angiogenesis/endothelial tube formation (TMSL/field; total master segment length per field averaged out of 5 assessed fields per condition, 16-hour stimulation with TNF-α, n=6) shown in **(B)**. Cells were first pre-treated with or without AP-1 blocker SR-11302 (10 nM) for 1 hour, followed by stimulation for 16 hours with or without 1 or 1000 pg/ml TNF-α in the presence or absence TNF-α blocking antibody Infliximab (100 ug/ml). Mann-Whitney-test with mean ± SEM, *P < 0.05 and **P < 0.01.

### EC Culture and Tube Formation Assay

The ECs (human umbilical vein derived endothelial cells EA.hy926 line 55) were provided by Dr. Cora-Jean Edgell (North Carolina State University Chapel Hill, US) (42–44), and found to be functionally similar to primary HUVECs and microvascular HMECs (PromoCell, Heidelberg, Germany) in key readouts relevant to this study (**Figure S1**). The ECs were cultured in MCDB131 (ThermoFisher, Darmstadt, Germany) supplemented with 5% FCS, 100 U/ml penicillin, 100 μg/ml streptomycin, and 2 mM L-glutamine. For the endothelial tube formation assay (34, 45) Matrigel (Corning, Tewksbury, MA) was poured onto a 96-well plate (50 ml per well) and solidified at 37°C for 30 minutes. The ECs were seeded (20.000 cells per well) onto Matrigel and cultured in MCDB131 basic medium with 0.5% FCS, supplemented with or without 5% (vol/vol) HSP, USP, or different types of uremic patient serum fractions from the MCO study, as described in in the figures legends. Tube networks were photographed using a Zeiss Axiovert 40 CFL microscope (Zeiss, Oberkochen, Germany), and five randomly selected fields from each well were analyzed for capillary length using ImageJ 1.43 software (National Institutes of Health, Bethesda, MD). Cell viability was assessed by quantifying mitochondrial activity with the water-soluble tetrazolium (WST-8) salt assay according to the manufacturer’s instructions (PromoCell) (17).

### Analyses of Gene Expression by Real-Time qRT–PCR

Gene expression was assessed with reverse transcription and quantitative real-time polymerase chain reaction (qRT-PCR) (17, 33, 34, 46, 47). Total RNA was extracted by using the PerfectPure RNA Cultured Cell Kit (5 Prime, Hamburg, Germany), its concentration and purity was estimated with a spectrophotometer (Nanodrop; Thermo Fisher Scientific), and the RNA reverse transcribed into cDNA with random hexamer primers, and qRT-PCRs performed on a 7500 Fast Block Real-Time PCR system (Applied Biosystems). The human primer sequences were composed as follows (**Table S2**): VEGF (GenBank NM_001171623.1): forward primer (5’-AAGGAGGAGGGCAGAATCAT-3’) and reverse primer (5’-ATCTGCATGGTGATGTTGGA-3’), c-FOS (NM_005252.3): forward (5’-AGGAGAATCCGAAGGGAAAG-3’) and reverse (5’-CTTCTCCTTCAGCAGGTTGG-3’), and β2-microglobulin (NM_004048.2): forward (5’-GTGCTCGCGCTACTCTCTCT-3’) and reverse (5’-ATCTGCATGGTGATGTTGGA-3’). The specificity of the qRT-PCR reaction was verified with melting curve analysis and the relative amount of transcript calculated with the cycle threshold method, using the Applied Biosystems 7500 System v.1.2.3 software and gene expression normalized relative to the endogenous reference gene β2-microglobulin (17).

### DNA Constructs, Plasmids, Transient Transfection and Luciferase Assays

Progressive VEGF 5’-deletion luciferase plasmid constructs (pLuc 2068, pLuc 1340, pLuc 318, and pLuc 102) were provided by A. Scholz (Charité) as reported previously (48), and checked for the correct length by restriction digestion. For transient transfection studies, the ECs were seeded into six-well culture plates at a density that allowed them to reach 70–80% confluence after 24 hours. Transfections were performed using the TurboFect transfection reagent (Fermentas, Darmstadt, Germany) according to the manufacturer’s instructions. Cells were transfected in the absence of serum with Turbofect/DNA at a ratio of 1 ml/0.33 mg. ECs were transfected with the VEGF reporter plasmid (0.2 µg/well) and co-transfected with the reference pRL-TK Renilla plasmid (0.02 µg/well). Luciferase activity was assessed using the dual-luciferase reporter assay system (Promega, Mannheim, Germany) according to the manufacturer’s protocol. Luciferase activity was measured using a microplate luminometer (Fluostar Optima, BMG Labtech, Ortenberg, Germany) and normalized to background levels of Renilla luciferase activity from co-transfected control vectors. The human VEGF promoter region -266 to -53 (**Figure S2**; GenBank NT_007592.15) was analyzed by the Software PROMO - prediction of transcription factor binding sites: (http://alggen.lsi.upc.es/cgi-bin/promo_v3/promo/promoinit.cgi?dirDB=TF_8.3) for the presence and location of potential transcription factor binding sites.

### Nuclear Extracts, Electrophoretic Mobility Shift Assay, and Immunoassays

Nuclear extracts were prepared using the NE-PER Nuclear and Cytoplasmic Extraction Kit and oligonucleotide probes labeled with Biotin 3’ End DNA Labeling Kit (ThermoFisher). For the EMSA (34), the following probe was used (promoter region provided in parentheses): AP-1 - 5’-CAGGCTTCACTGAGCGTCCGCAG-3’ (-141 to -164). Each EMSA binding mixture (20 µl) contained 5 µg of nuclear extract, 20 fmol of labeled double-stranded probe, 1 µg of poly (deoxyinosinicdeoxycytidylic) acid, and 2 µl of 10 x reaction buffer, and was incubated at room temperature for 30 min. Protein–DNA complexes were analyzed by electrophoresis in 6% non-denaturing polyacrylamide gels and visualized using a LightShift Chemiluminescent EMSA Kit (ThermoFisher). Concentrations of VEGF and the soluble receptors sTNF-R1 and -R2 were measured with commercially available DuoSet ELISA kit (DY293B, DY225 and DY726; respectively, R&D Systems; Minneapolis, MN, US) (37). The TNF-α protein concentrations were measured using human TNF-α Antibody Pair Kits according to the manufacturer’s instructions (ThermoFisher) and explained elsewhere (34). All assays were designed and performed as per manufacturer’s instructions. Cell extracts were prepared as described earlier (49), electrophoresed on sodium dodecyl sulfate-polyacrylamide gels and analyzed by Western blotting using antibodies against GAPDH (Hytest, Turku, Finland), the target protein, and secondary peroxidase-conjugated IgG (Dianova, Hamburg, Germany) (**Table S3**). The bands were visualized with an Enhanced Chemiluminescence Detection System (Thermo Scientific) and Image J 1.43 software.

### Statistical Analysis

Data are expressed as mean ± SEM. Non-parametric data are presented as medians. Statistical analysis and visualization was performed using GraphPad Prism (GraphPad^®^, San Diego, US) and R (version 3.5.1). Analyses of multiple variables were performed by one-way analysis of variance with Student–Newman–Keuls post- or Kruskal–Wallis with Müller–Dunn post-test. A P-value < 0.05 was considered statistically significant.

## Results

### Study Design and Experimental Layout

This is a follow-up study to identify the molecular and biological mechanisms of action (MoA) associated with the beneficial anti-inflammatory shift reported with the use of novel improved molecular cut-off dialyzers in the PERCI-II trial (**Figures 1A, B**, **2A**). The clinical study design and clinical outcomes are described in detail in this prior article (28).

The goal of the current study was to explore the effect of “Expanded Hemodialysis Therapy” on ECs, in particular endothelial activation and maladaptation, and its possible causal link to uremia and inflammation. For this purpose, we first established an *in vitro* exposure model of ECs with uremic serum samples obtained from chronic HD patients during the PERCI-II-MCO trial (**Figures 2B–G** **)**, employing VEGF expression and production as markers for endothelial activation and endothelial tube formation as a functional readout. This was followed by mechanistic validation, employing healthy and uremic serum pools (HSP and USP, please see **Figure 4**) and selective blocking strategies and promoter studies, to decipher the underlying cellular signaling events (**Figures 3** **–** **6**).

The PERCI-II patients underwent two different dialysis regimes (A and B) (**Figure 2A**), employing different combinations of high-flux (HF) or medium cut-off (MCO) dialysis membranes, with the crossover trial design and the different trial stages depicted to the right. In the dialysis regimen A (HF, MCO, HF, HF): following a 4-weeks wash-in phase with HF membranes, the patients were first dialyzed with MCO membranes for four weeks, then followed by a 12-week HF phase composed of 2 stages (**Figure 2A** **upper panel**, and **Figures 2B, D**, and **F**). In contrast, in the dialysis regimen B (HF, HF, MCO, MCO): following a similar 4-weeks wash-in phase with HF membranes, the patients were dialysed for 4 weeks with HF dialysis, followed by 12 weeks of MCO dialysis composed of 2 stages (**Figure 2A** lower panel, and **Figures 2C, E, G**).

### Hemodialysis With Improved MCO Dialyzers Normalizes Endothelial VEGF Production and Angiogenesis Upon Uremic Serum Exposure of ECs *In Vitro*

First of all, serum samples from PERCI-II patients starting with four weeks of MCO dialysis did not induce an increase in VEGF expression or protein production in ECs, but exposure to serum from patients undergoing another four weeks of HF dialysis led to a strong induction of VEGF expression and protein release (P<0.05 to P<0.01, **Figures 2B, D** **)**, with a further rise in VEGF release after a total of 12 weeks on HF dialysis (P<0.001, **Figure 2D**).

In contrast, exposure of ECs to serum samples collected after four weeks of HF dialysis initially led to an increased VEGF expression (P<0.01, 1.75-fold increase, **Figure 2C**) and production (P<0.001, 2-fold, **Figure 2E**), but switching to MCO dialysis resulted in strongly reduced VEGF expression and production (P<0.05 and P<0.001, **Figures 2C, E** **)**. After 12 weeks of MCO dialysis there was no difference to VEGF baseline levels.

Concomitantly, *in vitro* angiogenesis was determined under the influence of uremic serum collected after dialysis with regimen A or B (**Figures 2F, G** **)**. An increased rate of angiogenesis was observed after HF dialysis (P<0.05), while incubation with healthy or MCO serum led to a reduction in endothelial tube formation (P<0.05). In analogy to the VEGF expression data, we found that this was a time-dependent effect, with a stronger reduction of angiogenesis after longer periods of expanded MCO dialysis therapy.

### Altered TNF-α/sTNF-R1-Ratio in Patient Serum Is Associated With a Beneficial Shift in MCO Serum Cytokine Levels *In Vivo*

More than 30 soluble analytes were screened with multiplex technology in patient serum obtained at different study time points (**Figure 2A**) (28). Unsupervised clustering heat-map analysis (**Figure 3A**) of different biomarkers (rows) and patients (columns) demonstrated a random distribution of regimen A and B in the “Pre-treatment” wash-in phase, as would be expected, since both regimen were similar at this stage (End of phase 0, both regimen HF; with random sequence of orange vs. green in upper left cluster “regimen” bar, **Figure 3A**).

Importantly, pre and post-treatment groups separated into two major clusters (“Pre/Post” bar, white vs. grey squares, **Figure 3A**), indicating a differential impact of HF vs. MCO dialysis, with considerable separation between patients undergoing regimen A and B (orange vs. green shows a stronger grouping/larger clustering in the group to the right), thereby identifying several molecular targets of the TNF-signaling pathway (e.g. TFN-α and sTNF-R1 and R2) to be of key importance for this separation (bottom right, red markings, **Figure 3A**).

Interestingly, we found a dialysis-time dependent shift in the TNF-α/sTNF-R1-ratio in the patient serum dialyzed with MCO membranes compared to HF-dialysis (**Figure 3B**), which was most evident for patients undergoing the longer 12-weeks period of MCO dialysis (P<0.05 at 12-weeks and P<0.05 and P<0.01 for the comparison of pooled 4 and 12-weeks data phase 1-3 HF vs. MCO). This shift resulted from a reduction in sTNF-R1 (P<0.05; **Figure 3B**, left panel), which was not the case for sTNF-R2 and its TNF-α/sTNF-R2-ratio, which only showed a minor shift in the same direction (**Figure 3B**, right panel).

In line with the above, we found a correlation between TNF-α/sTNF-R1-ratio in the patient serum and endothelial VEGF production and angiogenic capacity upon exposure of ECs to corresponding serum *in vitro* (P=0.01 and P=0.08; **Figure 3C** left panel), which was not observed for the respective TNF-α/sTNF-R2-ratio (P=0.94 and P=0.63; **Figure 3C** right panel), indicating that sTNF-R1 is of crucial importance.

As recently shown by our group in the corresponding clinical report 27, MCO-dialyzers have an improved molecular cut-off (molecular sieving coefficient) and thereby promote improved uremic toxin removal and reduction of chronic inflammation. We thus reanalysed and substratified our preliminary data according to the individual study phases and found that patients undergoing MCO dialysis in particular for a longer duration (12 vs. 4 weeks), show a beneficial anti-inflammatory shift in multiple soluble mediators (P<0.05 to P<0.001; e.g. sVCAM and sFAS P<0.05 to P<0.001 at 8-12 weeks, and IL10, CXCL1 and Rantes P<0.05 at 12 weeks, **Figures 3D** and **S1**).

### Elevated TNF-α and VEGF-Levels in Uremic Serum From HD Patients and Endothelial VEGF Production Upon Exposure to Uremic Serum

To study the detailed molecular signaling events underlying endothelial maladaptation, we employed representative healthy and uremic serum pools (HSP and USP) (**Figure 4A**). First of all, key uremic mediators were found to be elevated in uremic serum: TNF-α (P<0.01; healthy 47.14 ± 6.57 vs. uremic 112.0 ± 16.18), sTNF-R1 (P<0.001; healthy 6.87 ± 0.75 vs. uremic 13.56 ± 1.24) and VEGF (P<0.001; healthy 113.0 ± 13.95 vs. uremic 331.3 ± 49.92). The serum levels of these factors were in a comparable range to prior HD studies when anticipating CKD-stage (21, 22).

To determine optimal assay conditions for our experiments (Optimal serum concentration and exposure time, e.g. used in **Figure 2**) we studied VEGF mRNA expression and protein production after incubation of ECs with HSP and USP (**Figures 4B, C** **)**. Kinetic profiling documented a differential peak of 1.75-fold increase in VEGF mRNA after 3-hours of serum incubation (P<0.001 at 3-hours and P<0.05 at 6- to 9-hours, **Figure 4B**, left panel) and maximal VEGF protein expression at 6-to 24-hours of incubation (all three P<0.01, **Figure 4B**, right panel) in response to 10% USP compared to 10% HSP. To also assess the ideal serum concentration for EC stimulation, we incubated the ECs with 1-20% serum, detecting a 1,5- to 2-fold increase in VEGF mRNA expression in response to 10-20% USP vs. HSP (P<0.05 to P<0.001, **Figure 4C**, left panel) and a 1.5- to 2-fold increase in VEGF protein secretion in response to 10-20% USP vs. HSP (all three P<0.05, **Figure 4C**, right panel).

Considering the optimal use of our valuable USP samples we decided to use 10% serum pool for all subsequent experiments, with detection of mRNA expression at 3-hours and protein secretion at 6-24-hours. In line with prior quantification of TNF-α and sTNF-R1 in patient serum, we observed no significant changes in the TNF-α/sTNF-R1-ratio when conducting serial dilutions of uremic serum (Range 1-20% as also used in the assays, data not shown), thus indicating stability of the TNF-α/sTNF-R1-ration during serial dilution.

### The Uremic Toxin TNF-α Mediates Endothelial VEGF Promoter Activation and Concomitant Induction of Maladaptive Angiogenesis *via* AP-1/c-FOS Signaling

In line with prior identification of TNF signaling (**Figure 3**), we studied whether proinflammatory interleukins in uremic serum are responsible for inducing VEGF expression, by adding IL-1 receptor antagonist Anakinra or anti-TNF-α blocking antibody Infliximab (**Figures 4D–E**). While antagonizing IL-1 with Anakinra failed to dampen the effect of uremic serum on VEGF production (**Figure 4D**), the blockade of TNF-α signaling with 10, 50, or 100 µg/mL Infliximab inhibited uremia-induced VEGF production in a dose-dependent manner (No blocking antibody P<0.01, 10 µg/mL P<0.05, and 50, or 100 µg/mL no significant difference compared to HSP, **Figure 4E**). We also observed lower EC viability upon incubation with USP compared to HSP (P<0.001, 59-46% vs. 83-85%, respectively, **Figure 4F**) and a USP dose-dependent reduction in EC viability from 59% (1% USP), to 50% (P<0.05; 15% USP) and 46% (P<0.001; 20% USP).

To substantiate the role of TNF-α in endothelial VEGF induction, we assessed whether incubation of ECs with TNF-α affects endothelial VEGF promoter activity (**Figure 5**). Indeed, we found a strong increase in full-length VEGF 2068 promoter activity when adding low concentrations of TNF-α (P<0.01; 1 pg/ml; **Figure 5A**), while very high TNF-α doses led to lower promoter activity. Accordingly, VEGF mRNA levels and rates of angiogenesis were increased after incubation with 1 pg/ml TNF-α (P<0.05, **Figures 5B, C** **)**, but decreased when adding 100 and 1000 pg/ml TNF-α (P<0.01 and P<0.001).

Next, we analyzed the VEGF promoter sequences responsive to TNF-α by employing ECs transiently transfected with step-wise 5’-deleted VEGF-A promoter-driven luciferase constructs (**Figure 5D**). We identified a TNF-dependent transcription factor binding-site between -267 and -52 bp upstream. In-silico promoter binding site analysis revealed three transcription factors (SOX10, AP-1, and NMZF-1), of which AP-1/cFOS was validated *via* gel shift assay as the specific VEGF promoter-binding site in ECs **(** **Figure 5E** **)**.

We quantified c-FOS mRNA expression relative to TNF-α concentration and the effect of sTNF-R1 on c-FOS mRNA expression in low and high TNF-α environment was assessed (**Figure 5F**). Incubation with 1 pg/ml TNF-α led to 2-fold upregulation of c-FOS (P<0.05), while increasing TNF-α to 1000 pg/ml resulted in lower c-FOS activity (P<0.05). In line with the bimodal relationship above (**Figures 5A–C**), in a low TNF-α environment (1pg/ml), increasing concentrations of sTNF-R1 reduced c-FOS induction (P<0.05), while in a high TNF-α environment, increasing levels of sTNF-R1 activated c-FOS (P<0.05).

Next, we linked the TNF-α- and s-TFN-R1-mediated regulation of AP-1/c-FOS-signaling to endothelial VEGF-expression and *in vitro* angiogenesis (**Figures 6A, B** **)**. Addition of 1 pg/ml TNF-α led to an increased VEGF-expression and angiogenesis (P<0.05), which was reduced to baseline by addition of c-FOS inhibitor SR-11302 and Infliximab (P<0.05 to P<0.01). In contrast, high TNF-α concentrations suppressed VEGF mRNA expression and angiogenesis (both P<0.01), which could be partially reversed by blocking AP-1/c-FOS (P<0.05) and completely reversed through the blockade of TNF-α with Infliximab (P<0.01).

In conclusion, endothelial VEGF expression and angiogenesis are strongly dependent on both the TNF-α and sTNF-R1 concentration in their environment and can be blocked by targeting TNF-α and AP-1/c-FOS signaling pathways.

## Discussion

Hemodialysis is a widely employed blood cleansing technique, but the use of conventional hemodialyzers is associated with a high cardiovascular morbidity and mortality resulting in part from the underlying chronic inflammation associated with this treatment. Thus, there is a high medical need to reduce inflammation and adverse cardiovascular events, which may be achieved with novel improved molecular cut-off MCO dialyzers in both, the chronic CKD/ESRD and the acute COVID-19 associated renal failure and RRT setting (**Figure 1A**) (6, 8, 9, 27).

In the current study, we have explored the molecular and biological mechanisms underlying this antiinflammatory shift by evaluating promising leads identified during the first screen (28). We found that uremic serum induces endothelial maladaptation and dysfunction through a signaling cascade, being triggered through TNF-α/sTNF-R1 signaling and transduced through AP-1/c-FOS signaling, thereby promoting maladaptive endothelial VEGF expression and angiogenesis (**Figure 1B**). Importantly, adjusting the permeability of the dialyzer by employing MCO membranes could abolish these detrimental signaling. The use of novel improved molecular cut-off MCO dialyzers shifted the TNF-α/sTNF-R1-ratio and inflammatory milieu in patient serum to ameliorate endothelial dysfunction.

Vascular disease and endothelial dysfunction are common in dialysis patients (20–22). One of the causes for this phenomenon is the retention of middle-sized proinflammatory molecules, which are closely linked to cardiovascular morbidity and mortality (8, 9, 24, 27). While the connection between inflammation and vascular smooth muscle cell calcification is already been well established (17), the detailed molecular, cellular, and biological interrelations between uremia, inflammation, and endothelial dysfunction remain widely unclear to date.

VEGF is a key regulator of angiogenesis in various settings, such as inflammation, and targeting VEGF has shown fist beneficial effects e.g. in vascular disease (50–52). In analogy to earlier studies by our group (33, 34), we here employed VEGF induction and angiogenesis as a readout parameter/marker for endothelial maladaptation and dysfunction (**Figure 1B**). Reports by multiple groups have shown an interrelation between HD-induced systemic VEGF-release, endothelial damage, and increased morbidity and mortality, with respect to CKD-stage (21–23, 32). Merino et al. have shown how different dialysis modalities affect the microinflammatory status, endothelial damage, and concomitant changes in VEGF levels (21). These were found to be increased in both, CKD patients without HD, and in patients undergoing peritoneal dialysis (PD), but highest in ESRD patients on chronic HD, compared to the healthy controls.

In addition, multiple groups reported an association between VEGF gene expression and circulating VEGF levels with inflammation and mortality in dialysis patients (22, 23, 32). VEGF levels are elevated in obesity and hypertension, with treatment of hypertension resulting in normalization of VEGF levels (53, 54). Thus, VEGF is as a valuable *in vitro* and *in vivo* marker of endothelial dysfunction (33, 34). VEGF is induced in ECs in response to stress, such as in inflammation and hypertension, and a major growth and survival factor for ECs that is essentially involved in angiogenesis. Sophisticated mechanistic *in vivo* studies by Domigan et al. have shown how autocrine VEGF production is essential for both the optimal function and survival of healthy endothelium (36). Prolonged endothelial stress by uremic toxins can result in compensatory endothelial maladaptation, VEGF overproduction, and functional exhaustion of its regenerative capacity, thereby leading to vascular disease and cardiovascular events, which may result in tissue ischemia, limb amputation and death (55). Our results show that VEGF is strongly upregulated in ECs in response to the uremic mediators found in serum from patients dialyzed with conventional HF dialyzers, as part of the early initiation and progression of uremia-induced endothelial dysfunction (**Figure 1B**). Importantly, we also show that this detrimental maladaptive process is reversible, since serum from patients treated with MCO induced less VEGF production and angiogenesis.

We hypothesized that the profile of uremic toxins/inflammation is altered/reduced during MCO dialysis, thereby resulting in reduced EC stress and activation. Indeed, unsupervised clustering heat-map analysis of the PERCI-II patient serum samples identified TNF superfamily members as being among the most strongly affected analytes impacted by the two different dialysis regimes, thereby identifying a potential relevance of TNF-α and its soluble receptors sTNF-R1 and sTNF-R2 in this setting. Multiple characteristic differences have been attributed to the two soluble TNF receptors. While circulating sTNF-R1 (55 kDa full-length form) is expressed in many cell types, sTNF-R2 (75 kDa) is expressed in more restricted fashion and the differences in their structure also suggest that they act in part through different downstream signaling pathways (40). While, sTNF-R1 binds equally well to the soluble and membrane-bound forms of TNF-α, sTNF-R2 has higher affinity for the membrane-bound form (56). In addition, soluble TNF receptors have also been implied to act as decoy-receptors for TNF-α (40). The ratio of TNF-α and sTNF-R1 has been described to be decisive in controlling the inflammatory activity of TNF-α, but also as an indirect marker of altered systemic inflammation within the TNF-family context (38–40). Indeed, we found during our mechanistic validation that TNF signaling takes a central role in controlling endothelial VEGF production and angiogenic maladaptation and that the TNF-α/sTNF-R1-ratio in uremic patient serum is indicative of endothelial dysfunction with its clinical relevance for the design of HD filters and adjunct optimal care in RRT (8, 20, 24, 27, 30, 38–41). This builds on earlier work by Dutch and Belgian research groups, who have studied the importance of the TNF-α/sTNF-R-ratio in the CKD/ESRD and hemodialysis setting (38–40).

Our manuscript focuses on readout of the TNFa, sTNF-R1/R2, AP1/cFOS, and VEGF signaling axis in the context of detrimental effects of uremia on microvascular ECs and amelioration thereof by employing novel dialysis filters with improved molecular cutoff. For better understanding to readers, we have focused on these main mediators, but in principle the list of studied markers could be expanded to many other markers indicative of endothelial maladaptation/injury/damage markers, such as endothelial E-Selectin (ELAM-1), ICAM-1, VCAM-1, IL-6, IL-8, Tie-2 and the production of reactive oxygen species (ROS), as outlines in other studies on endothelial inflammation (37, 46, 57–59).

Prior studies by Naserian and coworkers have shown that the TNF-TNF-R2 axis plays a distinct role in VEGF production by bone marrow derived mesenchymal stromal cells (MSCs) and endothelial progenitor cells (EPCs), which are an *in vivo* reservoir for endothelial repair in the periphery. Here, the TNF-TNF-R2 axis may be involved in the triggering of immunosuppressive effects and thus also be of interest for clinical use (59). In our HD setting, we found a more predominant relevance of TNF-TNF-R1 signaling over TNF-TNF-R2 axis signaling in the induction of VEGF in the whole-blood environment and in the *ex vivo* culture experiments involving microvascular ECs and healthy vs. patient-derived uremic serum from the different HD regimens (phases). The modulatory aspect of the two employed dialysis regimens is mainly due to differential cutoff properties on the blood-interphase in the medium cut-off range, which leads to the specific differential elimination/retention of various proinflammatory mediators, but also many other medium sized molecular components that accumulate in blood of uremic patients (28). Thus, the recent findings by Naserian et al. and our current study may be complementary, since they may be reflective of different underlying *in vivo* tissue compartments, pathology/inflammatory status of the studied clinical indications, and the corresponding treatment regimes. Interestingly, this may also differ between blood circulation and extravascular tissue environment (40) (e.g. in the extravascular tissue ECM and interstitial tissue spaces) and should thus be studied in more detail in the future.

We found in our promoter activation and signaling studies that VEGF production in ECs was induced by TNF-α through AP-1/c-FOS-mediated activation of the VEGF promoter, thus providing new molecular targets for ameliorating uremia-induced inflammation. Indeed, TNF-α has been characterized as a critical molecule in uremia-induced vascular disease and that it can promote both, vascular calcification (17) and endothelial dysfunction (24, 38–40). Thus, treatments aiming to reduce vascular and chronic inflammatory diseases, such as “Expanded Hemodialysis Therapy”, may be of great value (28, 29). This is in line with prior studies demonstrating that uremia-induced pro-calcifying effects could be reduced effectively with MCO dialysis (60, 61). As recently emphasized by Ronco and Reis (6), in addition to the value of MCO in the chronic CKD/ESRD RRT-setting, the beneficial systemic anti-inflammatory effects of MCO dialysis may also be of interest to ameliorate the acute, potentially fatal inflammation in RRT-dependent COVID-19 patients, which has now lead to the initiation of first clinical studies to test MCO in the COVID-19 RRT setting.

## Conclusions and Study Limitations

We here investigated the detailed molecular signaling events underlying uremic serum and in particular TNF superfamily member-induced endothelial maladaptation and dysfunction. We first of all identified TNF family members as critical regulators in endothelial activation. We also demonstrate that any detrimental effects can be effectively ameliorated with expanded MCO hemodialysis therapy in patients. The most relevant clinical observation is the positive modulation of new dialysis membranes with improved porosity on the capacity of uremic serum to ameliorate endothelial dysfunction and altered angiogenesis. Importantly, although the uremic serum was derived from a randomized controlled trial, all experiments involving ECs were done in *in vitro* experiments and thus need to be interpreted carefully. Trials focusing on clinical endpoints that aim to examine *in vivo* endothelial function may provide an avenue for future research. Furthermore, in addition to TNF family members, a number of other molecular targets, such as VCAM and free light chains, were also strongly modulated with MCO dialysis and may thus be of interest for further studies.

## Data Availability Statement

The raw data supporting the conclusions of this article will be made available by the authors, without undue reservation.

## Ethics Statement

The studies involving human participants were reviewed and approved by Ethics Committees of the Martin-Luther-University Halle-Wittenberg and the Charité Berlin. The patients/participants provided their written informed consent to participate in this study.

## Author Contributions

Writing, review and editing: RC, GM, JK-M, JW, HM, OR, DD, K-UE, RS, and DZ. Experiments and investigation: RC, CL, LC, HZ, LE, KW, MG, RF, and DZ. Conception and Supervision: RC, GM, JW, HM, OR, DD, K-UE, RS, and DZ. All authors contributed to the article and approved the submitted version.

## Funding

This manuscript is a follow-up of a published clinical trial: PERCI-IIMedium- Cut-Off (MCO), which is registered at ClinicalTrials.gov NCT02084381 https://clinicaltrials.gov/ct2/show/NCT02084381) (28). The current manuscript focuses on the mechanistic side studies that aim to verify promising biological leads identified in the prior clinical trial. The lab research was funded by academic grants through BMBF, DFG, BCRT/BSRT and EU-Project Grants. The 2.1 Million Euro funding for the prior PERCI-II clinical study came from the German Ministry for Education and Research (BMBF, Project Codes 1313N11786, 13N11787, 13N11788, and 13N11789) and was headed on the academic side by Prof. Dr. Schindler and Dr. Daniel Zickler at Charité and Prof. Dr. Girndt in Halle. G.M.’s contributions were made possible by funding from the German Federal Ministry for Education and Research (BMBF) and German Research Foundation (DFG) through the Berlin Institute of Health (BIH)-Center for Regenerative Therapies (BCRT) and the Berlin-Brandenburg School for Regenerative Therapies (BSRT, GSC203), respectively, and in part by the European Union’s Horizon 2020 Research and Innovation Program under grant agreements No 733006 (PACE) and 779293 (HIPGEN).

## Conflict of Interest

The authors declare that the research was conducted in the absence of any commercial or financial relationships that could be construed as a potential conflict of interest.

## Publisher’s Note

All claims expressed in this article are solely those of the authors and do not necessarily represent those of their affiliated organizations, or those of the publisher, the editors and the reviewers. Any product that may be evaluated in this article, or claim that may be made by its manufacturer, is not guaranteed or endorsed by the publisher.

## Supplementary Material

Supplementary Figure S1 Endothelial cell line comparison for key assay readout parameters studied in this manuscript in addition to verification of typical morphological and functional parameters of ECs. (The figure can be found on the next page.). (A) Three different endothelial cell (EC) lines, human umbilical cord derived EA.hy929, and two EC lines purchased from PromoCell, HUVEC (PromoCell, macrovascular) and HMEC (PromoCell, microvascular), were stimulated with either 10% uremic or healthy human serum pool (USP vs. HSP) or TNF-a (1pg/ml) vs. resting control and assayed in parallel for their: (1) VEGF mRNA expression (AU; arbitrary units; 3-hour stimulation; upper panel; n=7) and (2) VEGF protein release (pg/ml; 24-hour stimulation; central panel; n=7), and (3) Endothelial tube formation *in vitro* (TMSL/field; 16-hour stimulation; lower panel; n=7), which demonstrated similar responsiveness to the different stimuli with only minor variation between the tree; (B) The surface expression of typical endothelial cell markers was assessed on HUVEC line EA.hy929 with flow cytometry as reported previously (47, 62–64), upon labeling of trypsin-detached cells in suspension with FITC-/PE-fluorochrome-conjugated monoclonal mouse-anti-human antibodies CD31-FITC (PECAM-1), CD62-FITC (E-Selectin), CD144-FITC (VE-Cadherin), and vWE-PE (von Willebrand Factor), all from Becton Dickinson, demonstrating surface staining for all four EC markers, as shown by histogram overlays of antibody labelled cells compared to respective unspecific isotype labelled control cells; and (C) In addition HUVEC line EA.hy929 demonstrated the typical functional characteristic of ECs to take up oxidised low-density protein (oxLDL) with the assay being conducted according to methodology reported earlier by our group (65), and the specificity of the uptake was demonstrated by competition with red fluorochrome labeled Dil-oxLDL (100 ug/ml) vs. unlabelled native oxLDL (100 ug/ml). In addition, a number of publications in high-ranking journals also demonstrate that EA.hy929 is well established in various cell culture model systems to study endothelial cells *in vitro* (42–44). Statistical evaluation was done with ANOVA, Mean ± SEM, with **P* < 0.05, ***P* < 0.01, and ****P* < 0.001.

Supplementary Figure S2 VEGF promoter sequence within c-FOS-binding site (-102 to -112 in Red). -267. GGGCGCGTGTCTCTGGACAGAGTTTCCGGGGGCGGATGGGTAATTTTCAGGCTGTGAACCTTGGTGGGGGTCGAGCTTCCCCTTCATTGCGGCGGGCTGCGGGCCAGGCTTCACTGAGCGTCCGCAGAGCCCGGGCCCGAGCCGCGTGTGGAAGG**GCTGAGGCTCG**CCTGTCCCCGCCCCCCGGGGCGGGCCGGGGGCGGGGTCCCGGCGGGGCGGAGCCATGCGCCCCCCCCTTTTTTTTTTAAAAGTCGGCTGGTAGCGGGGAGGatcgcggaggcttggggcagccgggtagctcggaggtcgtggcgctgggg+50

Supplementary Table 1 Demography of serum donors for uremic and healthy serum pool. **Abbreviations S1:** BMI, body mass index. CV risk factors, cardiovascular risk factors.

Supplementary Table 2 Sequences of primers used in quantitative-real-time-PCR analysis. **Abbreviations S2:** VEGF, Vascular Endothelial Growth Factor; B2M, beta-2 microglobulin; c-FOS, cellular FBJ Osteosarcoma oncogene; AP-1, activator protein-1; and Oligo, oligonucleotide.

Supplementary Table 3 Antibodies and reagents used for EMSA and western blot. **Abbreviations S3:** AP-1, activator protein-1; c-FOS, cellular FBJ Osteosarcoma oncogene; and GAPDH, glyceraldehyde 3-phosphate dehydrogenase.
