# Supplementary material for: Expanded Hemodialysis Therapy Ameliorates Uremia-Induced Systemic Microinflammation and Endothelial Dysfunction by Modulating VEGF, TNF-α and AP-1 Signaling
Source: Front Immunol. 2021 Nov 11;12:774052. doi: 10.3389/fimmu.2021.774052 (PMC8632537; doi:10.3389/fimmu.2021.774052)
Supplement: Supplementary Figure S1 — Endothelial cell line comparison for key assay readout parameters studied in this manuscript in addition to verification of typical morphological and functional parameters of ECs. (The figure can be found on the next page.). (A) Three different endothelial cell (EC) lines, human umbilical cord derived EA.hy929, and two EC lines purchased from PromoCell, HUVEC (PromoCell, macrovascular) and HMEC (PromoCell, microvascular), were stimulated with either 10% uremic or healthy human serum pool (USP vs. HSP) or TNF-a (1pg/ml) vs. resting control and assayed in parallel for their: (1) VEGF mRNA expression (AU; arbitrary units; 3-hour stimulation; upper panel; n=7) and (2) VEGF protein release (pg/ml; 24-hour stimulation; central panel; n=7), and (3) Endothelial tube formation in vitro (TMSL/field; 16-hour stimulation; lower panel; n=7), which demonstrated similar responsiveness to the different stimuli with only minor variation between the tree; (B) The surface expression of typical endothelial cell markers was assessed on HUVEC line EA.hy929 with flow cytometry as reported previously (47, 62–64), upon labeling of trypsin-detached cells in suspension with FITC-/PE-fluorochrome-conjugated monoclonal mouse-anti-human antibodies CD31-FITC (PECAM-1), CD62-FITC (E-Selectin), CD144-FITC (VE-Cadherin), and vWE-PE (von Willebrand Factor), all from Becton Dickinson, demonstrating surface staining for all four EC markers, as shown by histogram overlays of antibody labelled cells compared to respective unspecific isotype labelled control cells; and (C) In addition HUVEC line EA.hy929 demonstrated the typical functional characteristic of ECs to take up oxidised low-density protein (oxLDL) with the assay being conducted according to methodology reported earlier by our group (65), and the specificity of the uptake was demonstrated by competition with red fluorochrome labeled Dil-oxLDL (100 ug/ml) vs. unlabelled native oxLDL (100 ug/ml). In addition, a number of publications in high-ranki [file DataSheet_1.pdf]

Supplementary Figure Legends, Figures and Tables

Expanded Hemodialysis Therapy Ameliorates Uremia-Induced Systemic Microinflammation and Endothelial Dysfunction by Modulating VEGF, TNF-α and AP-1 Signaling

Figure S1: Endothelial cell line comparison for key assay readout parameters studied in this manuscript in addition to verification of typical morphological and functional parameters of ECs. (The figure can be found on the next page.)

(A) Three different endothelial cell (EC) lines, human umbilical cord derived EA.hy929, and two EC lines purchased from PromoCell, HUVEC (PromoCell, macrovascular) and HMEC (PromoCell, microvascular), were stimulated with either 10% uremic or healthy human serum pool (USP vs. HSP) or TNF-a (1pg/ml) vs. resting control and assayed in parallel for their: (1) VEGF mRNA expression (AU; arbitrary units; 3-hour stimulation; upper panel; n=7) and (2) VEGF protein release (pg/ml; 24-hour stimulation; central panel; n=7), and (3) Endothelial tube formation *in vitro* (TMSL / field; 16-hour stimulation; lower panel; n=7), which demonstrated similar responsiveness to the different stimuli with only minor variation between the tree; **(B)** The surface expression of typical endothelial cell markers was assessed on HUVEC line EA.hy929 with flow cytometry as reported previously [47, 62-64], upon labeling of trypsin-detached cells in suspension with FITC-/ PE-fluorochrome-conjugated monoclonal mouse-anti-human antibodies CD31-FITC (PECAM-1), CD62-FITC (E-Selectin), CD144-FITC (VE-Cadherin), and vWE-PE (von Willebrand Factor), all from Becton Dickinson, demonstrating surface staining for all four EC markers, as shown by histogram overlays of antibody labelled cells compared to respective unspecific isotype labelled control cells; and **(C)** In addition HUVEC line EA.hy929 demonstrated the typical functional characteristic of ECs to take up oxidised low-density protein (oxLDL) with the assay being conducted according to methodology reported earlier by our group [65], and the specificity of the uptake was demonstrated by competition with red fluorochrome labeled Dil-oxLDL (100 ug/ml) vs. unlabelled native oxLDL (100 ug/ml). In addition, a number of publications in high-ranking journals also demonstrate that EA.hy929 is well established in various cell culture model systems to study endothelial cells *in vitro* [42-44]. Statistical evaluation was done with ANOVA, Mean ± SEM, with $*P < 0.05$, $**P < 0.01$, and $***P < 0.001$.

Figure S1:

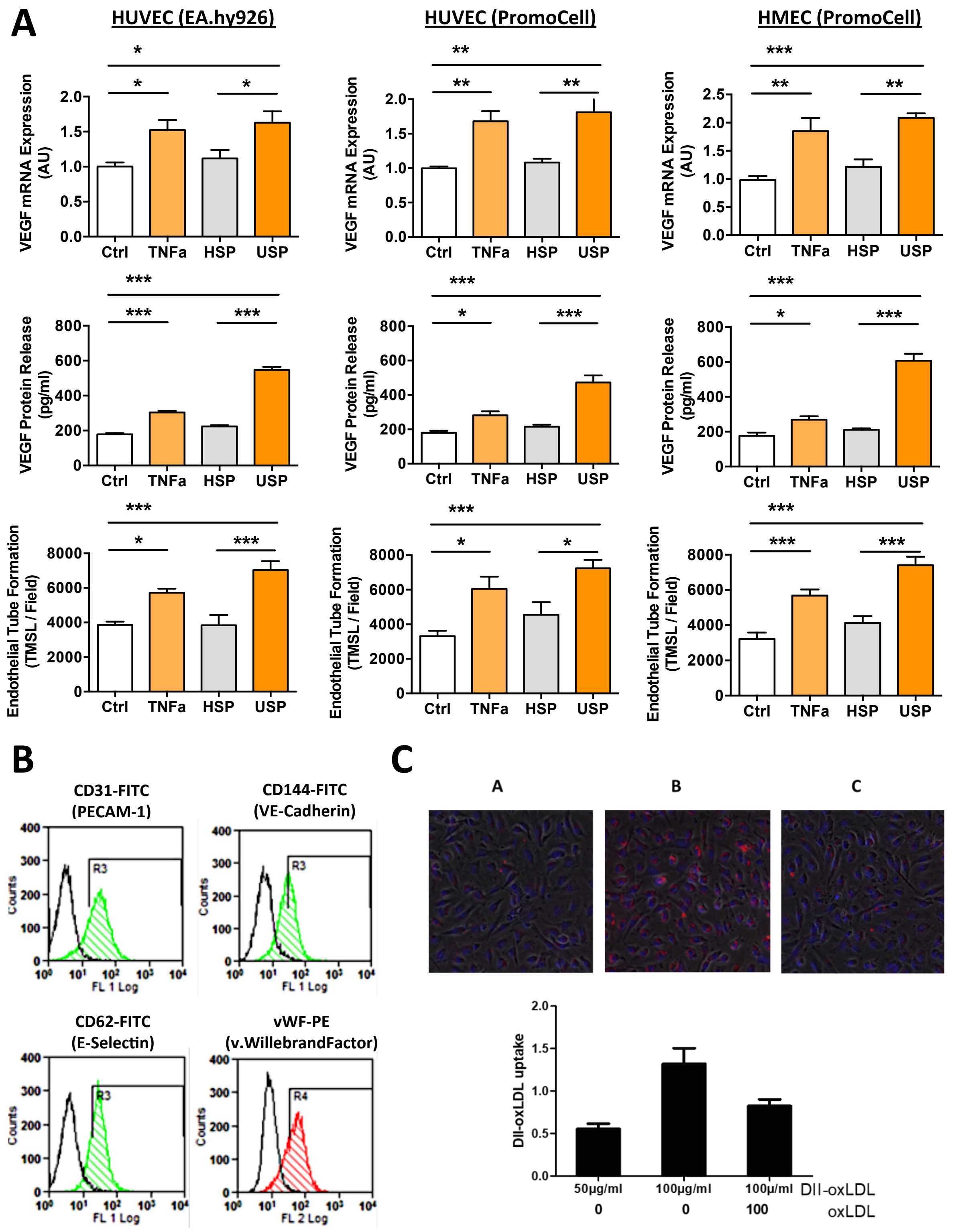

A
HUVEC (EA.hy926)
HUVEC (PromoCell)
HMEC (PromoCell)
VEGF mRNA Expression (AU)
VEGF Protein Release (pg/ml)
Endothelial Tube Formation (TMSL / Field)
Ctrl
TNFa
HSP
USP
B
CD31-FITC (PECAM-1)
CD144-FITC (VE-Cadherin)
CD62-FITC (E-Selectin)
vWF-PE (v.WillebrandFactor)
Counts
FL 1 Log
FL 2 Log
R3
R4
C
A
B
C
DiI-oxLDL uptake
50µg/ml
100µg/ml
100µ/ml
DiI-oxLDL
0
0
100
oxLDL

Figure S2: VEGF promoter sequence within c-FOS-binding site (-102 to -112 in Red)

-267

GGGCGCGTGTCTCTGGACAGAGTTTCCGGGGGCGGATGGGTAATTTTCAGGCTGTGAACCTTGGTGGGGGTCGAG

CTTCCCCTTCATTGCGGCGGGCTGCGGGCCAGGCTTCACTGAGCGTCCGCAGAGCCCGGGCCCGAGCCGCGTGTG

GAAGG**GCTGAGGCTCG**CCTGTCCCCGCCCCCCGGGGCGGGCCGGGGGCGGGGTCCCGGCGGGGCGGAGCCATGCG

CCCCCCCCTTTTTTTTTTAAAAGTCGGCTGGTAGCGGGGAGGatcgcggaggcttggggcagccgggtagctcgg

aggtcgtggcgctgggg+50

Table S1: Demography of serum donors for uremic and healthy serum pool.

**Demography**	**Uremic dialysis population** (n=20)	**Healthy control group** (n=14)
Age (years)	57 ±15.5	27 (±5.7)
Male, n (%)	13 (63 %)	9 (64%)
BMI	26.2 ±4.8	24 (±3.2)
**CV risk factors**		
Diabetes	4 (20 %)	0 (0%)
Hypertension	13 (75%)	0 (0%)
Smoking	7 (35 %)	4 (29%)

Abbreviations S1: BMI, body mass index. CV risk factors, cardiovascular risk factors.

Table S2: Sequences of primers used in quantitative-real-time-PCR analysis.

Gene	Sequence Sense Primer 5'→ 3'	Sequence Antisense Primer 3'→ 5'
**VEGF**	AAGGAGGAGGGCAGAATCAT	ATCTGCATGGTGATGTTGGA
**B2M**	GTGCTCGCGCTACTCTCTCT	CGGCAGGCATACTCATCTTT
**c-FOS**	AGGAGAATCCGAAGGGAAAG	CTTCTCCTTCAGCAGGTTGG
**AP-1 Oligo**	CAGGCTTCACTGAGCGTCCGCAG	CAGGCACTCGAGGCCTCA GACATCTCCAGTCC

Abbreviations S2: VEGF, Vascular Endothelial Growth Factor; B2M, beta-2 microglobulin; c-FOS, cellular FBJ Osteosarcoma oncogene; AP-1, activator protein-1; and Oligo, oligonucleotide.

Table S3: Antibodies and reagents used for EMSA and western blot.

Antibody	Target Antigen	Host	Code	Company	Dilution
**AP-1 / c-FOS**	AP-1 / c-FOS complex	Rabbit	#36201	Abcam	1:500
**GAPDH**	GAPDH protein	Mouse	#5G4	Hytest	1:50000
**2nd Antibody Anti-Mouse**	Mouse antigen	Donkey	#715035150	Dianova	1:5000
**2nd Antibody Anti-Rabbit**	Rabbit antigen	Donkey	#711035152	Dianova	1:10000

Abbreviations S3: AP-1, activator protein-1; c-FOS, cellular FBJ Osteosarcoma oncogene; and GAPDH, glyceraldehyde 3-phosphate dehydrogenase.
